# Tobacco Use Among Middle and High School Students — United States, 2011–2016

**DOI:** 10.15585/mmwr.mm6623a1

**Published:** 2017-06-16

**Authors:** Ahmed Jamal, Andrea Gentzke, S. Sean Hu, Karen A. Cullen, Benjamin J. Apelberg, David M. Homa, Brian A. King

**Affiliations:** ^1^Office on Smoking and Health, National Center for Chronic Disease Prevention and Health Promotion, CDC; ^2^Center for Tobacco Products, Food and Drug Administration.

Tobacco use is the leading cause of preventable disease and death in the United States; nearly all tobacco use begins during youth and young adulthood (*1*,*2*). Among youths, use of tobacco products in any form is unsafe (*1*,*3*). CDC and the Food and Drug Administration (FDA) analyzed data from the 2011–2016 National Youth Tobacco Surveys (NYTS) to determine recent patterns of current (past 30-day) use of seven tobacco product types among U.S. middle (grades 6–8) and high (grades 9–12) school students. In 2016, 20.2% of surveyed high school students and 7.2% of middle school students reported current tobacco product use. In 2016, among current tobacco product users, 47.2% of high school students and 42.4% of middle school students used ≥2 tobacco products, and electronic cigarettes (e-cigarettes) were the most commonly used tobacco product among high (11.3%) and middle (4.3%) school students. Current use of any tobacco product did not change significantly during 2011–2016 among high or middle school students, although combustible tobacco product use declined. However, during 2015–2016, among high school students, decreases were observed in current use of any tobacco product, any combustible product, ≥2 tobacco products, e-cigarettes, and hookahs. Among middle school students, current use of e-cigarettes decreased. Comprehensive and sustained strategies can help prevent and reduce the use of all forms of tobacco products among U.S. youths (*1*–*3*).

NYTS is a cross-sectional, voluntary, school-based, self-administered, pencil-and-paper questionnaire administered to U.S. middle and high school students. A three-stage cluster sampling procedure was used to generate a nationally representative sample of U.S. students attending public and private schools in grades 6–12. This report uses data from six NYTS waves (2011–2016). Sample sizes and response rates for 2011, 2012, 2013, 2014, 2015, and 2016 were 18,866 (72.7%), 24,658 (73.6%), 18,406 (67.8%), 22,007 (73.3%), 17,711 (63.4%), and 20,675 (71.6%), respectively.

Participants were asked about current use of cigarettes, cigars, smokeless tobacco,* e-cigarettes,^†^ hookahs (water pipes used to smoke tobacco),^§^ pipe tobacco,^¶^ and bidis (small imported cigarettes wrapped in a leaf). Current use for each product was defined as use on ≥1 day during the past 30 days. “Any tobacco product use” was defined as current use of one or more tobacco products, and “≥2 tobacco product use” was defined as current use of two or more tobacco products.** “Any combustible tobacco product use” was defined as current use of cigarettes, cigars, hookahs, pipe tobacco, and/or bidis.

Data were weighted to account for the complex survey design and adjusted for nonresponse; national prevalence estimates, 95% confidence intervals, and population estimates were computed and rounded down to the nearest 10,000. Current use estimates for 2016 are presented for any tobacco product, any combustible tobacco product, ≥2 tobacco products, and each tobacco product individually, by selected demographics for each school type (high school and middle school). Results were assessed for the presence of linear and quadratic trends during 2011–2016, adjusting for race/ethnicity, sex, and school grade.^††^ T-tests were performed to examine differences between findings in 2015 and 2016. For all analyses, p-values <0.05 were considered statistically significant.

In 2016, 20.2% of high school students (estimated 3.05 million) reported current use of any tobacco product, including 9.6% (1.44 million; 47.2% of current tobacco product users) who reported current use of ≥2 tobacco products. Among high school students, e-cigarettes were the most commonly used tobacco product (11.3% of current users), followed by cigarettes (8.0%), cigars (7.7%), smokeless tobacco (5.8%), hookahs (4.8%), pipe tobacco (1.4%), and bidis (0.5%) (Table). Males reported higher use of any tobacco product, ≥2 tobacco products, cigars, smokeless tobacco, and pipe tobacco than did females. E-cigarettes were the most commonly used tobacco product among non-Hispanic white (13.7%) and Hispanic (10.3%) high school students, whereas cigars were the most commonly used tobacco product among non-Hispanic black high school students (9.5%).

**TABLE:** Estimated percentage of middle and high school students who used tobacco products in the past 30 days, by product,* school level, sex, and race/ethnicity — National Youth Tobacco Survey, United States, 2016

Tobacco product	Sex % (95% CI)		Race/Ethnicity % (95% CI)				Total	
	Female	Male	White, non-Hispanic	Black, non-Hispanic	Hispanic	Other, non-Hispanic	% (95% CI)	Estimated no. of users^†^
**High school students**								
Electronic cigarettes	9.5 (7.8–11.5)	13.1 (11.4–14.9)	13.7 (11.9–15.7)	6.2 (4.8–7.9)	10.3 (8.2–12.8)	5.4 (3.6–8.0)	**11.3 (9.9–12.9)**	**1,680,000**
Cigarettes	6.9 (5.4–8.8)	9.1 (7.6–11.0)	9.9 (8.2–11.8)	3.9 (2.9–5.3)	6.4 (4.9–8.4)	4.8 (3.1–7.6)	**8.0 (6.7–9.6)**	**1,180,000**
Cigars	5.6 (4.3–7.2)	9.0 (8.6–11.2)	7.9 (6.5–9.6)	9.5 (7.8–11.5)	7.2 (5.7–9.1)	3.7 (2.4–5.7)	**7.7 (6.6–8.9)**	**1,130,000**
Smokeless tobacco	3.3 (2.4–4.4)	8.3 (6.8–10.1)	7.4 (6.0–9.1)	2.1 (1.5–3.1)	4.4 (3.4–5.7)	3.8 (2.1–6.8)	**5.8 (4.8–7.0)**	**860,000**
Hookah	5.1 (4.1–6.3)	4.5 (3.8–5.4)	4.5 (3.7–5.4)	4.1 (3.2–5.3)	6.4 (4.8–8.3)	3.4 (2.1–5.5)	**4.8 (4.1–5.7)**	**700,000**
Pipe tobacco	0.9 (0.7–1.2)	1.8 (1.5–2.4)	1.4 (1.1–1.8)	1.2 (0.7–2.0)	1.2 (0.9–1.8)	—^§^	**1.4 (1.1–1.7)**	**190,000**
Bidis	0.3 (0.2–0.6)	0.7 (0.5–0.9)	0.4 (0.2–0.7)	—	0.6 (0.4–1.1)	—	**0.5 (0.3–0.7)**	**70,000**
**Any tobacco product^¶^**	**17.0 (14.9–19.3)**	**23.5 (21.3–25.8)**	**23.0 (20.7–25.6)**	**16.4 (14.1–18.9)**	**18.3 (15.8–21.0)**	**11.3 (8.7–14.5)**	**20.2 (18.4–22.3)**	**3,050,000**
≥2 tobacco products**	7.8 (6.3–9.7)	11.4 (9.9–13.0)	11.3 (9.6–13.2)	6.1 (5.2–7.3)	8.9 (7.1–11.2)	5.0 (3.2–7.7)	**9.6 (8.3–11.1)**	**1,440,000**
Any combustible tobacco product^††^	12.4 (10.7–14.4)	15.3 (13.7–17.1)	15.1 (13.1–17.3)	12.9 (11.0–15.1)	12.9 (11.1–14.9)	8.1 (5.9–11.1)	**13.8 (12.3–15.5)**	**2,080,000**
**Middle school students**								
Electronic cigarettes	3.4 (2.7–4.3)	5.1 (4.2–6.1)	3.7 (3.0–4.7)	4.0 (2.6–6.0)	5.6 (4.3–7.4)	—	**4.3 (3.7–4.9)**	**500,000**
Cigarettes	1.8 (1.3–2.5)	2.5 (1.8–3.4)	1.9 (1.4–2.6)	—	2.5 (1.8–3.5)	—	**2.2 (1.7–2.7)**	**250,000**
Cigars	1.7 (1.1–2.4)	2.7 (1.9–3.9)	1.4 (0.9–2.2)	4.5 (2.8–7.1)	2.8 (1.9–4.2)	—	**2.2 (1.7–2.9)**	**260,000**
Smokeless tobacco	1.5 (0.9–2.4)	3.0 (2.2–4.0)	2.1 (1.5–3.0)	—	3.0 (2.1–3.4)	—	**2.2 (1.6–3.1)**	**260,000**
Hookah	1.9 (1.5–2.5)	2.1 (1.5–2.9)	0.9 (0.6–1.4)	2.8 (1.8–4.4)	3.7 (3.0–4.7)	—	**2.0 (1.6–2.5)**	**230,000**
Pipe tobacco	0.6 (0.3–1.0)	0.8 (0.5–1.3)	—	—	1.7 (1.1–2.6)	—	**0.7 (0.5–1.0)**	**70,000**
Bidis	—	0.4 (0.2–0.7)	—	—	0.6 (0.4–1.1)	—	**0.3 (0.2–0.5)**	**30,000**
**Any tobacco product^¶^**	**5.9 (4.9–7.3)**	**8.3 (6.8–9.9)**	**5.9 (4.7–7.3)**	**7.5 (5.5–10.1)**	**9.5 (7.5–11.8)**	**—**	**7.2 (6.1–8.4)**	**850,000**
≥2 tobacco products**	2.5 (1.8–3.4)	3.6 (2.7–4.7)	2.3 (1.7–3.0)	3.0 (2.0–4.3)	4.5 (3.3–6.1)	—	**3.1 (2.5–3.8)**	**360,000**
Any combustible tobacco product^††^	3.9 (3.0–5.0)	4.6 (3.4–6.2)	2.9 (2.2–3.7)	5.8 (4.0–8.3)	6.1 (4.7–7.9)	—	**4.3 (3.5–5.2)**	**510,000**

**Abbreviation**: CI = confidence interval.

* Past 30-day use of electronic cigarettes was determined by asking, “During the past 30 days, on how many days did you use electronic cigarettes or e-cigarettes?” Past 30-day use of cigarettes was determined by asking, “During the past 30 days, on how many days did you smoke cigarettes?” Past 30-day use of cigars was determined by asking, “During the past 30 days, on how many days did you smoke cigars, cigarillos, or little cigars?” Past 30-day use of hookahs was determined by asking, “During the past 30 days, on how many days did you smoke tobacco in a hookah or waterpipe?” Smokeless tobacco was defined as use of chewing tobacco, snuff, dip, snus, and/or dissolvable tobacco products. Past 30-day use of smokeless tobacco was determined by asking the following question regarding chewing tobacco, snuff, and dip: “During the past 30 days, on how many days did you use chewing tobacco, snuff, or dip?,” and the following question for use of snus and dissolvable tobacco products: “In the past 30 days, which of the following products did you use on at least one day: snus, dissolvable tobacco products?.” Responses from these questions were combined to derive overall smokeless tobacco use. Past 30-day use of pipe tobacco and bidis were determined by asking, “In the past 30 days, which of the following products have you used on at least one day: pipe filled with tobacco (not waterpipe), bidis (small brown cigarettes wrapped in a leaf)?”

^†^ Estimated total number of users is rounded down to the nearest 10,000 persons.

^§^ Data are statistically unreliable because samples size was <50 or relative standard error was >0.3.

^¶^ Any tobacco product use is defined as use of any tobacco product (electronic cigarettes, cigarettes, cigars, smokeless tobacco, hookahs, pipe tobacco, and/or bidis) on at least one day in the past 30 days.

** ≥2 tobacco product use is defined as use of two or more tobacco products (electronic cigarettes, cigarettes, cigars, smokeless tobacco, hookahs, pipe tobacco, and/or bidis) on at least one day in the past 30 days.

^††^ Any combustible tobacco use defined as use of cigarettes, cigars, hookahs, pipe tobacco, and/or bidis on at least one day in the past 30 days.

Among middle school students, 7.2% (0.85 million) reported current use of any tobacco product, and 3.1% (0.36 million; 42.4% of current tobacco users) reported current use of ≥2 tobacco products (Table). Among middle school students, e-cigarettes were the most commonly used tobacco product (4.3%), followed by cigarettes (2.2%), cigars (2.2%), smokeless tobacco (2.2%), hookahs (2.0%), pipe tobacco (0.7%), and bidis (0.3%). Among males, current use of any tobacco product was 8.3%, and among females, was 5.9%. Hispanics reported higher use of any tobacco product, use of ≥2 tobacco products, and use of hookahs than did non-Hispanic whites (Table). 

Among all high school students, current use of any tobacco product did not change significantly from 2011 (24.2%) to 2016 (20.2%); however, a nonlinear decrease occurred in current use of any combustible tobacco product (21.8% to 13.8%), and ≥2 tobacco products (12.0% to 9.6%) during this time (Figure 1). By product type, nonlinear increases occurred for current use of e-cigarettes (1.5% to 11.3%) and hookahs (4.1% to 4.8%) (p for trend <0.05); however, a linear decrease occurred in current use of cigarettes (15.8% to 8.0%), cigars (11.6% to 7.7%), and smokeless tobacco (7.9% to 5.8%), and a nonlinear decrease occurred in current use of pipe tobacco (4.0% to 1.4%) and bidis (2.0% to 0.5%) (p<0.05 for trend) (Figure 1). During 2011–2016, among middle school students, a linear decrease occurred in current use of any combustible tobacco products (6.4% to 4.3%), cigarettes (4.3% to 2.2%), cigars (3.5% to 2.2%), and pipe tobacco (2.2% to 0.7%) (p for trend <0.05), whereas no significant linear or quadratic trends were observed for current use of any tobacco product or ≥2 tobacco products (Figure 2). A nonlinear increase occurred in current use of e-cigarettes (0.6% to 4.3%), and a linear increase occurred for current use of hookahs (1.0% to 2.0%) (p for trend <0.05).

**FIGURE 1:**
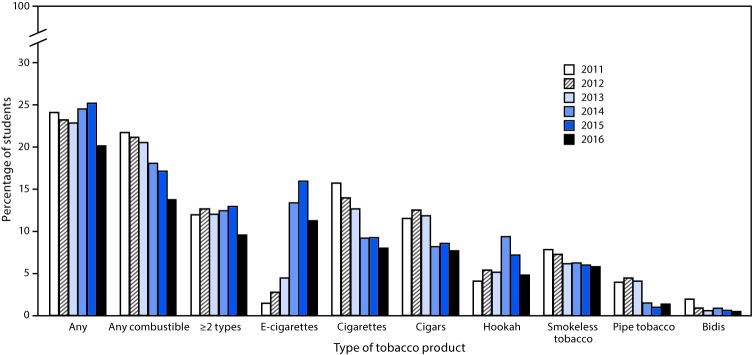
Estimated percentage of high school students who currently use any tobacco products,* any combustible tobacco products,**^†^** ≥2 tobacco products,^§^ and selected tobacco products — National Youth Tobacco Survey, United States, 2011–2016**^¶^**,**,**^††^** * Any tobacco product use is defined as past 30-day use of electronic cigarettes, cigarettes, cigars, hookahs, smokeless tobacco, pipe tobacco and/or bidis. ^†^ Any combustible tobacco use is defined as use of cigarettes, cigars, hookahs, pipe tobacco, and/or bidis on at least one day in the past 30 days. ^§^ ≥2 tobacco product use is defined as past 30-day use of two or more of the following tobacco products: electronic cigarettes, cigarettes, cigars, hookahs, smokeless tobacco, pipe tobacco, and/or bidis. ^¶^ From 2015 to 2016, a significant decrease in use of any tobacco product, any combustible tobacco product, ≥2 tobacco products, electronic cigarettes, and hookahs was observed (p<0.05). ** During 2011–2016, use of electronic cigarettes and hookahs exhibited a nonlinear increase (p<0.05). Use of cigarettes, cigars, and smokeless tobacco exhibited a linear decrease (p<0.05). Any combustible tobacco use, pipe tobacco, and bidis exhibited a nonlinear decrease (p<0.05). There was a nonlinear change during this time in the use of ≥2 types of tobacco products (p<0.05). No significant trend in current use of any tobacco product was observed during 2011–2016. ^††^ Beginning in 2015, the definition of smokeless tobacco included chewing tobacco/snuff/dip, snus, and dissolvable tobacco because of limited sample sizes for individual products; this definition was applied across 2011–2016 for comparability purposes. In previous reports (National Youth Tobacco Survey 2014 and earlier) smokeless tobacco included only chewing tobacco/snuff/dip; snus and dissolvable tobacco were reported as separate products.

**FIGURE 2:**
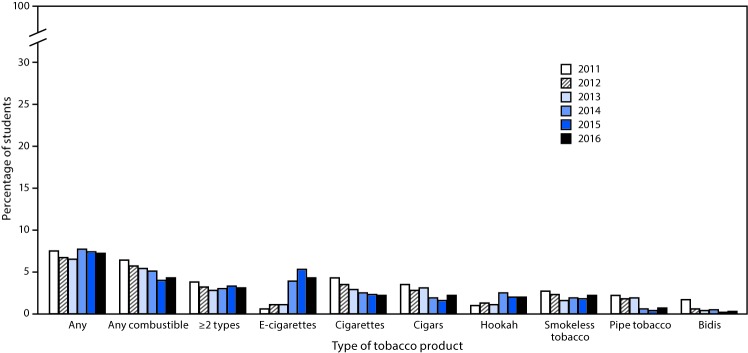
Estimated percentage of middle school students who currently use any tobacco products,* any combustible tobacco product,† ≥2 tobacco products,^§^ and selected tobacco products — National Youth Tobacco Survey, 2011–2016^¶^,**,^††^ * Any tobacco product use is defined as past 30-day use of electronic cigarettes, cigarettes, cigars, hookahs, smokeless tobacco, pipe tobacco and/or bidis. ^†^ Any combustible tobacco use is defined as use of cigarettes, cigars, hookahs, pipe tobacco, and/or bidis on at least one day in the past 30 days. ^§^ ≥2 tobacco product use is defined as past 30-day use of two or more of the following tobacco products: electronic cigarettes, cigarettes, cigars, hookahs, smokeless tobacco, pipe tobacco, and/or bidis. ^¶^ From 2015 to 2016, a significant decrease in use of electronic cigarettes was observed (p<0.05). ** During 2011–2016, electronic cigarette use exhibited a nonlinear increase (p<0.05). Hookah use exhibited a linear increase (p<0.05). Use of any combustible tobacco, cigarettes, cigars, and pipe tobacco exhibited a linear decrease (p<0.05). Bidi use exhibited a nonlinear decrease (p<0.05). Smokeless tobacco use exhibited a nonlinear change over this time period (p<0.05). No change in current use of any product or ≥2 types of products was observed during 2011–2016. ^††^ Beginning in 2015, the definition of smokeless tobacco included chewing tobacco/snuff/dip, snus, and dissolvable tobacco because of limited sample sizes for individual products; this definition was applied across 2011–2016 for comparability purposes. In previous reports (National Youth Tobacco Survey 2014 and earlier) smokeless tobacco included only chewing tobacco/snuff/dip; snus and dissolvable tobacco were reported as separate products.

During 2015–2016, among high school students, decreases occurred in the use of any tobacco product (25.3% to 20.2%), any combustible tobacco product (17.2% to 13.8%), ≥2 tobacco products (13.0% to 9.6%), e-cigarettes (16.0% to 11.3%), and hookahs (7.2% to 4.8%) (p<0.05). Among middle school students, e-cigarette use decreased from 5.3% in 2015 to 4.3% in 2016 (p<0.05). Among middle and high school students, use of other tobacco products, including cigarettes, cigars, smokeless tobacco, pipe, and bidis, did not change significantly during 2015–2016.

## Discussion

During 2015–2016, the use of any tobacco product, any combustible tobacco product, ≥2 tobacco products, e-cigarettes, and hookahs declined among high school students, and e-cigarette use declined among middle school students. This is in contrast to prior recent years, when declines in the reported use of cigarettes and cigars occurred alongside increases in the use of other tobacco products, including e-cigarettes and hookahs, resulting in no change in the use of any tobacco product during 2011–2016. In 2016, an estimated 3.9 million U.S. middle and high school students currently used any tobacco product, with 1.8 million reporting current use of ≥2 tobacco products. Among youths, symptoms of nicotine dependence are increased in multiple tobacco product–users compared with single product–users (*4*).

Tobacco prevention and control strategies at the national, state, and local levels likely have contributed to the reduction in use of certain tobacco products, including e-cigarettes, among youths in recent years (*2*). Efforts to address youths’ use of tobacco products include youth access restrictions, smoke-free policies that include e-cigarettes, and media campaigns warning about the risks of youth tobacco product use. For example, since February 2014, FDA’s first national tobacco public education campaign, The Real Cost, has broadcasted tobacco education advertising designed for youths aged 12–17 years; the campaign was associated with an estimated 348,398 U.S. youths who did not initiate cigarette smoking during February 2014–March 2016 (*5*). Continued implementation of these strategies can help prevent and further reduce the use of all forms of tobacco product among U.S. youths (*1*–*3*).

The findings in this report are subject to at least three limitations. First, NYTS only recruited students from public and private schools; therefore, the findings might not be generalizable to youths who are being home-schooled, have dropped out of school, or are in detention centers. Second, data were self-reported; thus, the findings are subject to recall and response bias. Finally, changes in the wording and placement of survey questions about certain products (e.g., e-cigarettes, hookahs, and pipe tobacco) during 2011–2016 might have had an impact on reported use. Despite these limitations, overall trends are generally similar to those found in other nationally representative surveys (*6*,*7*).

Sustained efforts to implement proven tobacco control policies and strategies are critical to preventing youth use of all tobacco products. Effective August 8, 2016, FDA finalized its deeming rule, which gave FDA jurisdiction over products made or derived from tobacco, including e-cigarettes, cigars, pipe tobacco, and hookah tobacco (*8*). Regulation of the manufacturing, distribution, and marketing of tobacco products by FDA, coupled with full implementation of comprehensive tobacco control and prevention strategies at CDC-recommended funding levels (*9*), could reduce youth tobacco product initiation and use (*1*,*2*,*9*). Strategies to reduce youth tobacco product use include increasing the price of tobacco products, protecting people from secondhand exposure to combustible tobacco smoke and e-cigarette aerosol, implementing advertising and promotion restrictions and national public education media campaigns, and raising the minimum age of purchase for tobacco products to 21 years (*9*,*10*). Continued monitoring of all forms of youth tobacco product use is critical to determine whether current patterns in use persist over time.

**Summary**

**What is already known about this topic?**

Tobacco use is the leading cause of preventable disease and death in the United States, and nearly all tobacco use begins during youth and young adulthood. Among youths, use of tobacco products in any form is unsafe.

**What is added by this report?**

In 2016, one in five high school students and one in 14 middle school students reported current use of a tobacco product on ≥1 of the past 30 days (3.9 million tobacco users). Moreover, 47.2% of high school students and 42.4% of middle school students who used a tobacco product in the past 30 days used ≥2 tobacco products. During 2015–2016, current use of electronic cigarettes (e-cigarettes) decreased among middle school students, and decreases in current use of any tobacco product, any combustible tobacco product, ≥2 tobacco products, e-cigarettes, and hookahs occurred among high school students. However, decreases in cigarette and cigar use during 2011–2016 were offset by increases in hookah and e-cigarette use, resulting in no significant change in any tobacco use. In 2016, e-cigarettes remained the most commonly used tobacco product among high (11.3%) and middle (4.3%) school students.

**What are the implications for public health practice?**

Sustained efforts to implement proven tobacco control strategies focusing on all types of tobacco products are critical to reduce tobacco product use among U.S. youths.

## Footnotes

* Beginning in 2015, the definition of smokeless tobacco included chewing tobacco/snuff/dip, snus, and dissolvable tobacco because of limited sample sizes for individual products (snus, dissolvable). In figures 1 and 2, this definition was applied across all years (2011–2016) for comparability purposes. The definition of smokeless tobacco in previously published reports (NYTS 2014 and earlier) included only chewing tobacco/snuff/dip, whereas snus and dissolvable tobacco were reported as separate products.

† In 2015 and 2016, current use of e-cigarettes was assessed by the question “During the past 30 days, on how many days did you use electronic cigarettes or e-cigarettes?” E-cigarette questions were preceded by an introductory paragraph. In 2016, this paragraph read: “The next thirteen questions are about electronic cigarettes or e-cigarettes. E-cigarettes are battery-powered devices that usually contain a nicotine-based liquid that is vaporized and inhaled. You may also know them as vape-pens, hookah-pens, e-hookahs, e-cigars, e-pipes, personal vaporizers or mods. Some brand examples are NJOY, Blu, Vuse, MarkTen, Logic, Vapin Plus, eGo, Halo.” A similar introductory paragraph preceded e-cigarette questions in 2015. In 2014, current use of e-cigarettes was assessed by the question “During the past 30 days, on how many days did you use e-cigarettes such as Blu, 21st Century Smoke, or NJOY?”; and in 2011 to 2013, e-cigarette use was assessed by the question “In the past 30 days, which of the following products have you used on at least one day?,” and the response option for e-cigarettes was “Electronic cigarettes or e-cigarettes such as Ruyan or NJOY.”

§ In 2016, current use of hookahs was assessed by the question “In the past 30 days, on how many days did you smoke tobacco in a hookah or waterpipe? Hookah questions were preceded by an introductory statement: “The next eight questions are about smoking tobacco in a hookah, which is a type of waterpipe. Shisha (or hookah tobacco) is smoked in a hookah.” From 2011–2015, current hookah use was assessed by the question “In the past 30 days, which of the following products have you used on at least one day?” Hookah was the fourth response option in 2015, the first response option in 2014, and was the fourth or fifth response option from 2011 to 2013.

¶ From 2014 to 2016, current use of tobacco pipes was assessed by the question “In the past 30 days, which of the following products have you used on at least one day?” and the response option for pipe tobacco was “Pipe filled with tobacco (not waterpipe).” Pipe tobacco was the second response option available in 2016, the fifth option in 2015, and the second option available in 2014. From 2011 to 2013, tobacco pipe use was assessed by the question “During the past 30 days, on how many days did you smoke tobacco in a pipe?”

** In 2015 and 2016, the definition of ≥2 tobacco product–use includes the updated definition of smokeless tobacco, thereby analyzing chewing tobacco/snuff/dip, snus, and dissolvable tobacco as a single tobacco product type compared with previously published NYTS reports, which analyzed chewing tobacco/snuff/dip, snus, and dissolvable tobacco as separate products.

†† A test for linear trend is significant if an overall statistically significant decrease or increase occurs during the study period. Data were also assessed for the presence of quadratic trends; a significant quadratic trend indicates that the rate of change accelerated or decelerated across the study period. Trends were only assessed when statistically stable data were available for all 6 years. A significant positive linear trend and nonsignificant quadratic trend signifies the presence of a linear increase; a significant negative linear trend and nonsignificant quadratic trends signifies the presence of a linear decrease; a significant positive linear trend and significant positive or negative quadratic trend signifies the presence of a nonlinear increase; a significant negative linear trend and significant positive or negative quadratic trend signifies the presence of a nonlinear decrease; a nonsignificant linear trend and significant positive or negative quadratic trend signifies the presence of a nonlinear change.
