# *Erratum:* Vol. 66, No. 23

**DOI:** 10.15585/mmwr.mm6628a8

**Published:** 2017-07-21

In the report, “Tobacco Use Among Middle and High School Students — United States, 2011–2016,” an error occurred in the Table. The prevalence estimate for cigar smoking among high school males should read “**9.9** (8.6–11.2).”

